# Body Mass Index Change as a Predictor of Biometric Changes following an Intensive Lifestyle Modification Program

**DOI:** 10.1155/2019/8580632

**Published:** 2019-03-24

**Authors:** David Drozek, Alexandria DeFabio, Randi Amstadt, Godwin Y. Dogbey

**Affiliations:** ^1^Department of Specialty Medicine, Ohio University Heritage College of Osteopathic Medicine, Athens, OH 45701, USA; ^2^Ohio University Heritage College of Osteopathic Medicine, Athens, OH 45701, USA; ^3^Cleveland Clinic, Cleveland, OH 44195, USA; ^4^School of Osteopathic Medicine, Campbell University, Buies Creek, NC 27506, USA

## Abstract

The initial benefits of lifestyle modification programs such as reduction in chronic and cardiovascular diseases (CVD) risk factors have been well documented. However, such positive effects may deteriorate over time following relapse into inactivity. Timely detection of weight regain leading to the deterioration of the accrued benefits could trigger early resumption of intensive lifestyle intervention. To date, no known cost-effective, noninvasive approach for monitoring long-term outcomes has yet been established. The purpose of this study was to determine if body mass index (BMI) change predicted changes in other CVD biometric markers during an intensive lifestyle modification program. This study was an observational, retrospective review of records of participants from the Complete Health Improvement Program (CHIP). Biomarker changes of participants in this community-based Intensive Therapeutic Lifestyle Modification Program (ITLMP) offered in Athens, Ohio, a rural Appalachian college town, between April 2011 and June 2017 were reviewed retrospectively. BMI, heart rate (Pulse), systolic blood pressure (SBP), diastolic blood pressure (DBP), and fasting blood levels of total cholesterol (TC), low-density lipoprotein (LDL), high-density lipoprotein (HDL), triglycerides (TG), and glucose (FBS) were monitored before and after program completion. Data were analyzed using a multivariate general linear model. The sample analyzed consisted of 620 participants (mean age of 52.3±13.0 years, 74.5% female). Controlling for age and gender, BMI change significantly predicted 5 out of the 8 biomarker changes measured [Wilk's *λ* = 0.939,* F*(8,526) = 4.29,* p* <.0001]. Specifically, a 1-point BMI decrease was associated with 4.4 units decrease in TC, 3.2 units in LDL, 5.3 units in TG, 2 units in SBP, and 1 unit in DBP (all* p* values < .05). These results suggest that change in BMI may be a useful predictor of change in other CVD biomarkers' outcomes during and after an ITLMP participation. Tracking BMI, therefore, could serve as a proxy measure for identifying regressing biomarker changes following participation in an ITLMP leading to a timelier reassessment and intervention. Future studies evaluating the value of BMI as a surrogate for highlighting overall cardiovascular health are warranted.

## 1. Introduction

Cardiovascular disease (CVD) is the leading cause of death in the United States [1]. Risk factors for CVD include dyslipidemia, hypertension, elevated body mass index (BMI), and diabetes [2–4]. Intensive therapeutic lifestyle modification programs (ITLMP) have been effective in improving all of these CVD risk factors [5–7]. One well studied ITLMP is the Complete Health Improvement Program (CHIP), which has demonstrated short-term effectiveness in improving many CVD risk factors [8, 9]. CHIP is an intensive, community-based lifestyle intervention program administered locally by a nonprofit organization, Live Healthy Appalachia located in Athens, Ohio. Another study, Look AHEAD, provided strong evidence in support of the efficacy of intensive lifestyle intervention (ILI), especially in Type 2 diabetes patients. It was observed that the ILI patients compared to a control group (that received standard treatment), across a four-year span of observation, registered desirable results regarding glycemic control, blood pressure reduction, lower triglycerides, and improvements in other CVD related risk factors [10].

The Appalachian region of the United States has consistently been associated with high morbidity and mortality resulting from chronic diseases such as heart disease and diabetes, lack of access to health care, and large numbers of uninsured people [11]. High levels of poverty, unemployment, inadequate housing and transportation, and limited education underpinned the often-undesirable health status of this population. Prior studies have demonstrated that CHIP was effective in reducing chronic disease and CVD risk factors in an Appalachian population [12, 13]. Such benefits were shown to have been independent of health-care payment source and worked across the socioeconomic strata, and were enhanced by participating in the program with another household member [14].

While the initial benefits of and adherence to lifestyle modification programs have been well documented, these positive effects can diminish over time without sustained intensive intervention efforts [10, 15]. If the onset of deterioration, usually evidenced by regain of lost weight and relapse into inactivity, could be readily detected early on, additional lifestyle modification intervention could be administered to mitigate the decay of the benefits. Indeed, the Look AHEAD study demonstrated that ILI yielded significant weight loss that was maintained by participants resulting in improved health outcomes. The rural impoverished population of much of Appalachia faces transportation barriers to reach distant health services, such as laboratories, possibly discouraging patients from follow up evaluation and treatment. Consequently, a cost-effective, convenient and noninvasive approach for monitoring long-term health outcomes among such medically underserved population would not only be compelling but desirable.

To determine the usefulness of a simple anthropometric measure such as BMI as a proxy to predict the health outcomes in an Appalachian population would be instructive and helpful to population health. Indeed, a study of Chinese Buddhist vegetarians suggested that BMI might be a good marker for CVD risk [16]. Therefore, the purpose of the present study was to determine if a change in body mass index (BMI) would be predictive of changes in other CVD biomarkers during an ITLMP in a rural Appalachian population.

## 2. Methods

### 2.1. Study Participants

Records of 620 participants from 26 different classes of the Complete Health Improvement Program (CHIP), a community-based ITLMP offered in Athens, Ohio, a rural Appalachian college town, between April 2011 and June 2017 were reviewed. The participants had varied socioeconomic backgrounds, being recruited via local media, health-care providers, and churches. They were members of the Athens community as well as communities from the surrounding areas—counties, suburbs, and townships. As part of the registration process for CHIP, participants were asked to sign an informed consent to give permission for the use of their deidentified aggregated data for research purposes. Data were stored on password-protected devices with restricted access to only approved CHIP administrators and study investigators. Approval for the study was obtained from the Ohio University Institutional Review Board.

### 2.2. CHIP Description

The CHIP intervention consisted of 16 to 18 two-hour group sessions that were provided over 4 to 12 weeks. The CHIP sessions were delivered by trained volunteers using standardized session materials produced by the CHIP administrators, the nonprofit organization Live Healthy Appalachia (LHA). A typical session consisted of an instructional video, group discussion, cooking demonstrations, and an exercise component [9]. The goal of CHIP was for participants to consume plant-based whole foods, such as minimally processed vegetables, fruits, whole grains, legumes, and nuts. This was done through fostering self-care and awareness of lifestyle habits. In fact, participants followed a self-monitoring regimen in terms of their lifestyle modifications. There was no direct monitoring of their adherence; trust and integrity were essential tenets of the program emphasized and conveyed to participants during recruitment and participation. Specifically, overall dietary fat was to be kept below 20% of total calories, daily intake of sugar less than 10 teaspoons, salt intake less than 2000 mg, cholesterol below 50 mg, and fiber intake to be 35 to 40 grams. Stress management techniques were taught and encouraged for daily use. Daily exercise of at least 30 minutes of moderate activity or 10,000 steps measured using a pedometer was encouraged. Strength training and resistance exercises were encouraged for 20-30 minutes, 2-3 days per week.

### 2.3. Inclusion/Exclusion Criteria

There were no formal inclusion/exclusion criteria except the timeline under review for participation. The data were captured by trained personnel working with the nonprofit organization (LHA) in Athens, Ohio.

### 2.4. Data Collection and Reporting

Biomarkers on which data were collected before and after completion of the program were BMI, as well as heart rate (Pulse), systolic blood pressure (SBP), diastolic blood pressure (DBP), and fasting blood levels of total cholesterol (TC), low-density lipoprotein (LDL), high-density lipoprotein (HDL), triglycerides (TG), and glucose (FBS). BMI was calculated as weight in kilograms divided by the square of height in meters squared [17]. Blood was collected via venipuncture by trained phlebotomists. The data were entered into a password-protected Microsoft Access database at the Live Healthy Appalachia (LHA) office by trained personnel within the office. The records were reviewed by trained medical students under the supervision of the personnel of LHA. Data on participants whose records fell within the period under consideration were collected.

All participants underwent the same program. No control group was involved. The study was based on retrospective review of program data. However, CHIP by its own design was patient-outcomes centered, so data were collected at baseline and after the completion of the program to gauge program effectiveness. Enrollment into CHIP was voluntary and was on a rolling basis, participants taking the sessions of a class together. CHIP collected baseline data whenever a participant initially enrolled in the program. Participants whose data were used in this study enrolled in CHIP at different times within the period under review. The same curriculum was provided and measurements taken on participants regardless of time of enrollment.

### 2.5. Statistical Analysis

Data were analyzed using a multivariate general linear model framework where the changes in the biomarkers were treated as a linear set of combined dependent variables with BMI as a predictor together with age and gender. Essentially, the functional specification of the model was* V* =* f *(BMI, Gender, Age), where *V* was a vector of a linear combination of the changes in the CVD biomarkers of interest as a linear function of BMI change, Gender, and Age of participants. Preliminary analyses of data were conducted to ensure that there were no data entry errors. Missing data were dropped from the analyses—they were not imputed, on variable-by-variable basis. Statistical significance was set at* p* ≤.05.

## 3. Results

The 620 participants constituting the sample had a mean age of 52.3 (±13.0 sd) with a range of 17-82 years. The composition of the sample of participants was predominantly female (74.5%)—approximately a 3:1 female-to-male ratio (Table 1).

As is typical of CHIP, all biomarkers, other than HDL, had positive short-term changes [8, 9]. The decrease in HDL in CHIP has been described and discussed before, and is not considered detrimental, but a compensatory response to lower LDL levels [12, 18] (Table 2).

Controlling for age and gender in the multivariate linear analysis, a unit BMI change significantly predicted changes in 5 out of the 8 biomarker changes measured [Wilk's *λ* = 0.939,* F*(8,526) = 4.29,* p *< .0001]. Specifically, a 1-point decrease was associated with 4.4 units decrease in TC, 3.2 units in LDL, 5.3 units in TG, 2 units in SBP, and 1 unit in DBP (all* p*-values <.05) (Table 3).

## 4. Discussion

The current study demonstrates that a reduction in BMI following an intensive lifestyle change intervention is associated with desired changes in levels of lipids and blood pressure. Obtaining evidence that demonstrated that BMI—a quick and inexpensive measure–could serve as a good proxy in predicting CVD risk factors in a medically underserved population is not only reasonable but compelling.

Generally, higher BMI is associated with higher total cholesterol, lower HDL, higher blood pressure, and diabetes, all critical biomarkers for cardiovascular disease [19]. Determining how much weight loss is needed for an individual to lower LDL to less than 100 mg/dl may be helpful to encourage participants to reach both their weight loss goal and reduction in their CVD risk. A meta-analysis by Zomer et al., focused on interventions that cause weight loss and their impact on cardiovascular risk factors, determined that a kilogram of weight loss significantly reduced SBP (−2.68 mmHg, 95% CI −3.37, −2.11), DBP (−1.34 mmHg, 95% CI −1.71, −0.97), LDL (−0.20 mmol L^−1^, 95% CI −0.29, −0.10), TG (−0.13 mmol L^−1^, 95% CI −0.22, −0.03), FBG (−0.32 mmol L^−1^, 95% CI −0.43, −0.22) and hemoglobin A1c (−0.40%, 95% CI −0.52, −0.28) over 6–12 months and that significant changes remained after 2 years for several risk factors [20].

Weight loss may not correlate with loss of fat mass. Cruz et al. found that favorable improvement in body composition may go undetected in almost a third of people whose weight remains the same and in another third of people who gain weight after attending a wellness center [21]. However, in a different study, a weekly self-reported weight checklist, turned out to be a simple yet valid and reliable tool for gauging adherence and desired outcomes in weight management. It provided relevant clinical information regarding weekly changes and related health outcomes in patients [22]. Self-monitored and reported weights after completion of an ITLMP could similarly be a useful tool to indicate early loss of adherence to program regimen and thus the return of chronic disease risk factors.

### 4.1. Limitations

This study is a retrospective study of changes of CVD biomarkers during CHIP. The changes in BMI after CHIP may not be a reliable indicator of change in other biomarkers. However, this preliminary evidence should be enough to warrant and encourage design and implementation of a randomized, controlled prospective study. Indeed, weight loss may or may not be healthy—weight loss occurs with cancer, chemotherapy, eating disorders and other chronic diseases. However, conscious weight loss associated with participation in an ITLMP may be an indicator of positive changes in other CVD risk factors.

The participants in this study were overwhelmingly women and could possibly skew the results, as men are known to improve CVD risk factors better with lifestyle change [23, 24]. The first reported CHIP intervention study, a hospital-based program conducted in Kalamazoo, Michigan (*n* = 288) demonstrated that males with the highest levels of TC at program entry (i.e., 240-279 mg/dL) experienced a 22% reduction in 30 days, whereas females with the highest levels of TC experienced a mean decrease of 11% [23]. These findings were consistent with those reported in an Australasian CHIP study wherein men showed improved responsiveness to reductions in chronic disease risk factors compared with women [24].

The participants enrolled voluntarily and thus constituted a self-selected sample. Hence, they more likely could be motivated than the population in general. Moreover, there could be a confounding of the results since no accompanying measures of adherence were collected. However, such confounding would mean that the results could have been better had those adherence metrics been measured and controlled for.

## 5. Conclusion

Change in BMI may be helpful as an important predictor of change in other CVD biomarkers during an ITLMP. Tracking of BMI could serve as a promising proxy variable for detecting adherence to weight management programs and predicting decay of initial health outcomes following participation in an intensive lifestyle modification program. Future studies that further evaluate and validate BMI as a surrogate for predicting outcomes of selected biomarkers after completion of an ITLMP are warranted.

## Figures and Tables

**Table 1:** Basic demographics of the participant sample.

Age, mean (± sd), years	52.3 (±13.0)
Gender, *n* (%)	

Male	158 (25.5)

Female	462 (74.5)

**Table 2:** Beginning and end averages of biomarkers with mean and percent change.

	Begin (Mean)	End (Mean)	Mean Change	% Change
TC (mg/dl)	187.2	167.83	19.37	+10.35
HDL (mg/dl)	49.13	45.44	3.69	+7.51
LDL (mg/dl)	113.08	98.1	14.98	+13.25
TG (mg/dl)	131.1	123.74	7.36	+5.61
FBS (mg/dl)	105.91	100.16	5.75	+5.43
SBP (mmHg)	128.66	122.66	6	+4.66
DBP (mmHg)	77.6	75.4	2.2	+2.84
Pulse (bpm)	74.8	71.3	3.5	+4.91
Weight (pounds)	200.07	193.51	6.56	+3.26
BMI (1 kg/m^2^)	32.1	31.03	1.09	+3.4

Abbreviations: total cholesterol (TC), high-density lipoprotein cholesterol (HDL), low-density lipoprotein cholesterol (LDL), triglyceride (TG), fasting blood glucose (FBS), systolic blood pressure (SBP), diastolic blood pressure (DBP), and body mass index (BMI).

**Table 3:** Significant changes of biomarkers associated with 1 kg/m^2^ change of BMI.

Biomarker	Magnitude of estimated change associated with 1 kg/m^2^ change of BMI	95% confidence interval (LL, UL)
TC, mg/dl	4.4	(2.27, 6.53)
LDL, mg/dl	3.2	(1.32, 4.98)
TG, mg/dl	5.3	(1.36, 9.21)
SBP, mmHg	2	(0.802, 3.23)
DBP, mmHg	1	(0.15, 1.86)

Abbreviations: total cholesterol (TC), low-density lipoprotein cholesterol (LDL), triglyceride (TG), systolic blood pressure (SBP), and diastolic blood pressure (DBP), body mass index (BMI), LL = Lower Limit and UL = Upper Limit of a 95% confidence interval.

## Data Availability

The raw data used to support the findings of this study are available from the corresponding author upon request.
